# Peptide Nucleic Acid-Based Biosensors for Cancer Diagnosis

**DOI:** 10.3390/molecules22111951

**Published:** 2017-11-11

**Authors:** Roberta D’Agata, Maria Chiara Giuffrida, Giuseppe Spoto

**Affiliations:** 1 Dipartimento di Scienze Chimiche, Università di Catania, Viale Andrea Doria 6, I-95125 Catania, Italy; dagatar@unict.it; 2 Consorzio Interuniversitario “Istituto Nazionale di Biostrutture e Biosistemi”, c/o Dipartimento di Scienze Chimiche, Università di Catania, Viale Andrea Doria 6, I-95125 Catania, Italy; mcgiuffrida@gmail.com

**Keywords:** peptide nucleic acid, biosensors, DNA, microRNA, liquid biopsy, cancer

## Abstract

The monitoring of DNA and RNA biomarkers freely circulating in the blood constitutes the basis of innovative cancer detection methods based on liquid biopsy. Such methods are expected to provide new opportunities for a better understanding of cancer disease at the molecular level, thus contributing to improved patient outcomes. Advanced biosensors can advance possibilities for cancer-related nucleic acid biomarkers detection. In this context, peptide nucleic acids (PNAs) play an important role in the fabrication of highly sensitive biosensors. This review provides an overview of recently described PNA-based biosensors for cancer biomarker detection. One of the most striking features of the described detection approaches is represented by the possibility to detect target nucleic acids at the ultra-low concentration with the capability to identify single-base mutations.

## 1. Introduction

Early cancer diagnosis and the frequent monitoring of cancer patients are key to achieving the target of reducing the mortality and improving the efficacy of pharmaceutical treatment [1,2]. Different methods are today applied to detect cancer, including biopsies, endoscopy, magnetic resonance imaging and blood tests [3]. Such diagnostic tools are not sensitive enough to screen patients at the very early stage of the disease progression. In addition, some of them can potentially introduce clinical risks for the patient, are costly and patient compliance with most of such procedures is variable given their invasive nature.

Different systems potentially acting as cancer biomarkers are present in the blood, including circulating tumour cells (CTCs) [4], membranous structures containing molecular biomarkers such as microvesicles and exosomes [5], circulating free nucleic acids [6] (i.e., circulating cell-free DNA, RNA and microRNA) [7] and proteins [8]. The study of such systems could provide a molecular spectrum of a tumour by avoiding the otherwise required sampling of tumour cells from the human body (tissue biopsy) [4,9]. On this basis, liquid biopsy [10] has emerged as a potential complement to traditional biopsy for early cancer diagnosis and tailor-made therapy [6].

The detection of cancer biomarkers circulating in the blood is a challenging task mostly due to the low concentration of biomarkers in early-stage patients. Thereby, the demand for new analytical methods for the sensitive and robust detection of molecular signatures of a tumour in the blood of patients has significantly increased over the last few years [11].

In this context, biosensors offer attractive alternatives to conventional platforms, thanks to presently available advanced possibilities for the sensitive target biosensing [12]. Biosensors are ideal platforms for constructing minimally invasive diagnostic tools able to provide molecular-level information to be used for implementation of personalised medicine [13,14].

Biosensors are analytical devices that incorporate a biological sensing element and are based on the conversion of molecular recognition events into a measurable signal generated by a transducer. Recent technological progress in microfluidics [15] and nanofabrication processes [16] offer new opportunities for the development of biosensing platforms for cost-effective, high-throughput, point-of-care (POC) diagnostics. In particular, the performance of biosensing platforms has benefited from the design of optimised surface chemistry [17] and detection schemes for the enhancement of the detected signal [18,19,20], and from the use of nanomaterials [21,22].

Nucleic acids are essential targets in cancer diagnostics and the platform used to identify them should be sensitive (limit of detection (LOD) down to picomolar–femtomolar concentrations) and selective enough to ensure accurate discrimination among biomolecules dispersed in biological fluids such as blood, urine and saliva [23]. DNA susceptibility to restriction enzymes, its reduced stability under pH and temperature conditions can limit the advantages in designed DNA biosensors [24].

In this scenario, significant benefits come from the use of nucleic acid analogues in nucleic acid biosensing [25]. DNA synthetic mimics such as peptide nucleic acids (PNAs) [26] or locked nucleic acids (LNAs) [27] have pushed biosensors to new perspectives allowing to achieve biosensing performances that are clinically relevant [28]. In particular, PNA displays many advantageous features in DNA targeting, such as its neutral charge and its higher stability and selectivity compared with nucleic acid analogues [29,30].

Here, we report an overview of recent advances in PNA-based biosensors with a specific emphasis on cancer diagnosis. Various sensing strategies are reviewed (Table 1) according to the potential they hold in providing a clinically relevant combination of sensitivity and selectivity. 

## 2. PNA-Based Biosensors

PNA [45] is a non-natural nucleic acid analogue whose backbone is composed by *N*-(2-aminoethyl)glycine motifs linked via peptide bonds [46]. The uncharged backbone makes PNA/DNA and PNA/RNA complexes more stable than the corresponding DNA/DNA and DNA/RNA systems. In addition, PNA exhibits chemical and thermal stability in conditions where DNA/RNA would undergo degradation [47]. PNAs are insensitive to ionic strength and pH changes and are resistant to enzymatic cleavage inside living cells. A single mismatch in PNA/DNA heteroduplexes decreases the melting temperature more than in DNA/DNA duplexes (about 15 °C compared with 4 °C) [48] thus demonstrating the higher level of selectivity of PNA compared with DNA [49].

PNAs are synthesized using standard peptide solid-phase synthetic protocols [50]. PNA oligomers are cleaved from the solid-state support using conventional chemical procedures, purified by reverse-phase high-performance liquid chromatography and characterised by mass spectrometry. PNAs are also commercially available, but the cost is still higher than DNA oligonucleotides. In general, pure PNAs are neutral compounds with a tendency for self-aggregation and limited water solubility. The latter properties are strongly dependent on pH and the buffer used. The introduction of charged groups in the PNA structure (e.g., a C-terminal lysine amide), improves PNAs’ general properties by minimising their tendency to aggregate [47].

The biophysical properties of PNA make it an excellent candidate for use in biosensing applications, particularly when used as the capture probe [51]. PNA probes capture complementary target sequences with higher efficiency than DNA probes thus contributing to enhance the assay sensitivity.

PNA hybridizes to complementary oligonucleotide sequences in agreement with the Watson-Crick base-pairing rules by establishing hydrogen bonds between complementary nucleobases. PNA exhibits superior hybridization features, and the different molecular structure of PNA/DNA duplexes compared with the DNA/DNA structure provide a range of chemical signatures that can be potentially detected after the hybridization, thus enabling the design of novel detection protocols. The use of neutral PNA probes offers new opportunities for the design of advanced biosensing platforms exploiting the variation of the charge conditions occurring after the hybridization of the complementary DNA or RNA negatively charged sequences. Similar approaches have been proposed in combination with the use of functionalized nanoparticles, redox indicators or the polymerization of oligomers to enhance the sensitivity of the assay [52].

PNA oligomers are able to invade dsDNA by a mechanism known as ‘strand invasion’ leading to the formation of a triplex structure [53]. Such a property is exploited for antisense or antigene strategy [54].

PNA oligomers have been used to detect tumour cells and to deliver small molecules acting as drugs [55,56]. However, PNA’s poor solubility in water and its low cellular uptake still represent essential obstacles to the use of unmodified PNAs for similar applications [57]. The chemical modification of the PNA backbone or its conjugation with charged peptides has been proposed to address such issues. A variety of different changes to the PNA structure have been investigated with the aim to enhance its binding properties, directionality in hybridization and selectivity. These include the displacement of glycine with a chiral amino acid. Such chemical modifications of the structure of PNAs provide different opportunities to modulate properties of PNA useful in facilitating the design of a biosensor.

Efforts have been paid in designing modified PNA structures showing enhanced binding features [58,59] to provide additional possibilities to modulate properties of PNA probes that facilitate the fabrication of new diagnostic biosensing platforms.

PNA is not a substrate for DNA polymerases and for this reason PNA clamps are used to inhibit PCR amplification of wild-type DNA templates [60]. The specificity of PNA-mediated PCR clamping is good enough to allow the discrimination of alleles differing by one single nucleotide polymorphism (SNP). PNA-clamp technology has been adopted in reactions known as ‘PCR Clamping’ and has been used to identify occult micrometastases in colorectal cancer (CRC) patients [61] and to detect KRAS point mutations in peripheral blood samples of CRC patients [62].

Numerous examples of PNA-based electrochemical [52], piezoelectric [63,64], surface plasmon resonance [65,66,67] and microarray [68,69] biosensors have been described. Here, we will focus only on biosensors using PNA probes to detect DNA or RNA analytes relevant to cancer diagnosis.

### 2.1. PNA-Based Biosensors for RNA Detection 

The growing list of non-coding RNA species involved in critical biological functions makes RNA an attractive target for molecular recognition [70,71]. In particular, prominent examples of RNAs implicated in several cancers are microRNA (miR) [72], messenger RNA (mRNA) [73], circulating RNAs [74] and long non-coding RNAs (lncRNAs) [75,76].

miRs are among the most studied RNAs present in eukaryotic cells. miRs are short (19–25 nucleotides long) RNA sequences acting as regulators [77,78]. They are involved in transcription and also translational repression and gene silencing. The complex formed when miR binds an enzyme, known as RISC (RNA-induced silencing complex), can interact with the complementary mRNA sequences. mRNA is then silenced after its enzymatic cleavage [79]. Mutations in miR sequences may result in a dysfunction or deregulation of their biogenesis, thus triggering a broad spectrum of diseases [80,81].

High-throughput detection of miRs is performed using microarrays [82,83,84]. Other methods for miR detection include reverse transcriptase (RT) PCR [85,86], surface-enhanced Raman scattering [87,88,89], droplet microfluidics [90] and surface plasmon resonance (SPR) [91].

The intrinsic small size, the sequence homology and the low concentration of miR make the detection of miR a challenging task [92]. When dealing with cancer diagnosis, miR biomarkers are upregulated or downregulated in distinct types of cancer, and some miRs are also linked to different cytogenetic abnormalities [93]. 

Chemical and physical properties of nanostructures and nanomaterials have often been exploited to enhance the sensitivity for miRs’ detection [94]. In particular, efforts have been paid to developing different kinds of functional nanomaterials, such as noble metal nanoparticles, magnetic nanoparticles, quantum dots, carbon-based nanomaterials, with the aim to push the LOD further down to picomolar [95]. In this context, the optical detection of fluorescence signals produced by labelled probes is often exploited. 

Graphene and graphene-like two-dimensional (2D) nanomaterials used in fluorescence resonance energy transfer assays hold great potential for use in biosensing [96]. In particular, graphene oxide (GO) represents the basis for the sensitive detection of miR in living cells based on the use of dye-labelled PNAs and nanosized GO (NGO) [31]. NGO quenches the fluorescence emitted by labelled PNA. In this case, PNA is preferred to DNA as the probe because of the lower fluorescence background generated and the more stable binding with NGO. The sensing approach is based on the recovery of the fluorescence of labelled PNA upon addition of miR. The use of three different PNA probes labelled with carboxy fluorescein (FAM)-PNA21, 6-carboxy-X-rhodamine (ROX)-PNA125b and cyanine 5 (Cy5)-PNA96, allowed the parallel detection of three different miRs expressed in cancer cell lines: miR-21, miR-125b and miR-96, respectively. The detection limit for the parallel detection of miRs was about 1 pM. 

Nanoporous metal-organic frameworks (MOFs) [97,98] exhibit an inherent fluorescence quenching capacity that can be exploited for miR detection by labelled PNA probes [32]. Labelled PNA bonded to the nano-MOF (NMOF) is released in the presence of target miR. The resulting hybridization between the PNA probe and complementary miR target allows the recovery of the fluorescence. In addition, in this case, the assay has been tested against three miRs expressed in cancer cell lines (miR-21, miR-96 and miR-125b) using complementary PNA probes labelled with different fluorophores. The lowest detected concentration of target miRs was about 10 pM.

Graphitic carbon nitride (g-C3N4) nanosheet can be used to design assays similar to those discussed below and exploiting the quenching of labelled PNA probes [96,99]. Carbon nitride nanosheet (CNNS) can be exfoliated from bulk g-C3N4 and directly dispersed in aqueous solution [100]. In addition, in this case, the recovery of the fluorescence emitted by labelled PNA probes adsorbed on CNNS when in contact with complementary miR sequences is used to assay miR in the complex medium [33]. 

Considerable attention is today devoted to the fabrication of biosensing devices using low-cost and flexible materials for applications also in resource-limited settings [101]. In this perspective, a poly(vinylidene fluoride) thin sheet impregnated with poly(3-alkoxy-4-methylthiophene) (PT) and modified with a PNA probe can be used for the optical detection of miR [34]. The assay has been designed to perform a naked eye detection of miR-21 associated with lung cancer. Different optical signatures are generated when the non-specific adsorption of miR on the polymeric sheet or the specific interaction with the complementary PNA probe are established. In particular, an orange fluorescence signal is generated when the triplex system PT–PNA–miR-21 is formed. The assay shows a linear correlation in the 10 nM–10 mM concentration range as well as a successful mismatch detection.

Cationic polythiophene derivatives can be used as the active layer for a quartz crystal microbalance (QCM) surface modification to detect miR-21 spiked in plasma samples [35]. Negatively charged miR adsorbs on cationic polythiophene. The specific capture of miRs in complex media can be performed using biotinylated PNA sequence complementary to the miR target. Avidin-coated nanoparticles have been used to bind the biotinylated PNA/miR complex that has been subsequently adsorbed on polythiophene-modified QCM surface for signal amplification and to yield responses at clinically relevant concentrations (400 pM). 

Standard protocols for nucleic acid detection very often include the amplification of the target sequence. PCR is the most widely used method that combines the polymerase action with thermal cycling to amplify low abundance target sequences. Isothermal amplification methods have emerged as a promising alternative to PCR that significantly simplifies the implementation of amplification methods in POC diagnostic devices [102]. The integration of isothermal methods in microfluidic apparatus reduces the risk of sample contamination and minimises the required sample volume [90,103]. Recently, researchers succeeded in establishing bladder cancer diagnosis via detection of miRs from urine samples using a dual-isothermal cascade assisted lateral flow assay strategy [36]. The assay strategy combines base stacking hybridization (BSH) with exponential isothermal amplification (EXPAR) [104] and PNA probe. EXPAR produces ssDNA copies at a constant temperature and can be combined with various biosensing platforms. BSH results from the stability associated with hybridization reactions wherein two strands hybridize in a contiguous tandem to a longer complementary ssDNA. EXPAR occurs only in the presence of the target miR sequence based on the BSH process. The sample solution with EXPAR-amplified ssDNA was adsorbed on the sample pad of a lateral flow strip. Then, AuNPs–DNA conjugate was dispensed on the conjugate pad of the strip onto which two biotinylated PNA probes (test and control) were previously immobilized. The accumulation of AuNPs–DNA conjugates on the PNA probe test line produced a characteristic red line. The assay detects miR-126, miR-182 and miR-152 extracted from urine samples of bladder cancer patients and healthy donors down to 0.6 fM. 

Several approaches exist for the electrochemical detection of nucleic acids. Many such methods adopt specific procedures for signal enhancement, often combining nanostructured materials, enzymes and, in some cases, PNA probes [105]. Impedimetric detection has repeatedly been used to develop sensitive assays for miR detection. Such methods exploit the uncharged nature of PNA probes to design assays using negative charges of hybridized miR to trigger processes leading to sensitive detection.

**Figure 1:**
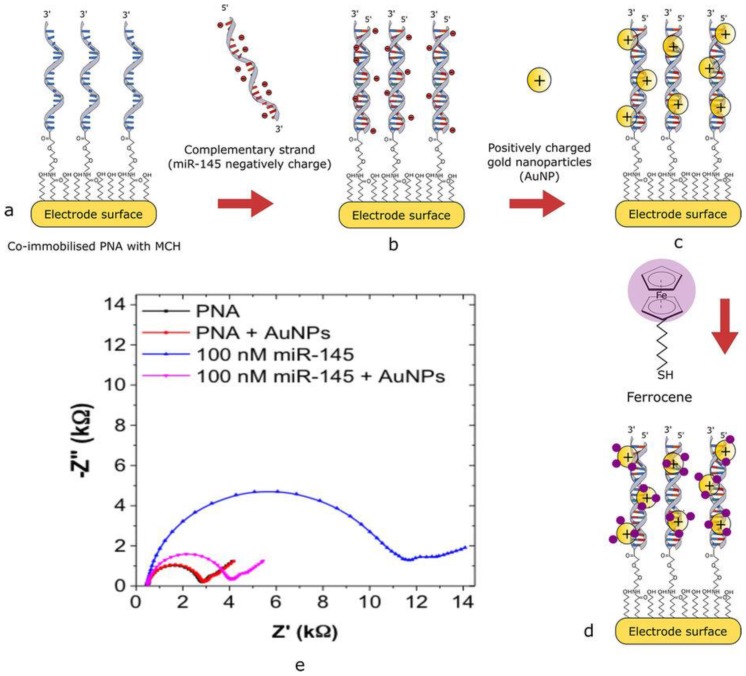
Schematic description of the dual mode electrochemical biosensor used to detect miR-145. After the thiolated PNA probes immobilisation (**a**) and miR-145 hybridization (**b**) an amplification strategy using positively charged gold nanoparticles was used (**c**). Thiolated ferrocene was adsorbed on AuNPs (**d**) to produce an electrochemical signal that was recorded using square wave voltammetry. (**e**) Typical features observed after the impedimetric detection (Nyquist plot) are shown. Reprinted from Ref. [37].

Jolly et al. introduced a dual-mode electrochemical biosensor using thiolated PNA probes immobilised on the sensor gold surface to detect miR-145 [37]. After PNA probe hybridization with the target, an amplification strategy taking advantage of the neutral charge of the PNA probe and using positively charged AuNPs was used in combination with impedimetric detection to monitor binding events without the need for any redox markers (Figure 1). An additional detection mode using thiolated ferrocene was used on the same sensor. Thiolated ferrocene immobilised on AuNPs adsorbed on the PNA–miR-145 complex produced an electrochemical signal that was recorded using square-wave voltammetry and that increased with miR-145 concentration. The dual-mode strategy allows detecting miR with a 0.37 fM LOD and a wide dynamic range (1 fM–100 nM).

Another example of an assay exploiting the uncharged nature of PNA to obtain a sensitive impedimetric detection of miR has been provided by producing a miR-guided deposition of polyaniline [38]. miR was first hybridized onto the PNA probe previously immobilised on a gold electrode. The negatively charged surface was then exposed to a mixture containing aniline, H_2_O_2_ and a G-quadruplex-hemin DNAzyme to obtain a hybridized miR-guided polymerisation of aniline. The formed poly-aniline film affected the electron-transfer power that was measured to determine the concentration of the target miR. 0.50 fM target miR was detected with this approach with the capability also to provide mismatch discrimination.

Graphene-based field-effect transistors (FETs) have been widely used to perform nucleic acid detection [106,107] including miR [39]. In the latter case, AuNPs were used to decorate the surface of the reduced GO deposited on the surface of the FET sensor. Then, PNA probes were immobilised on AuNPs and utilised for miR let-7b detection. A 10 fM concentration of the target sequence was detected with discrimination of point mutation (let-7c) and unrelated sequences (miR-21). miR let-7b was also spiked to human serum at 1 fM and 10 fM concentration with a successful detection of the target species.

### 2.2. PNA-Based Biosensors for DNA Detection

Cancer is linked to mutations that accumulate stepwise in genomic DNA, thus triggering a network of processes responsible for carcinogenesis [108]. For this reason, clinicians use the analysis of the tumour-linked genetic mutations for diagnostic and prognostic purposes. The detection of mutations whose presence is linked to the reduced efficacy of specific drugs able to slow the tumour progression is also used in patient follow-up and therapy efficacy assessment.

The detection of mutations present in DNA available from tumour cells (CTC) and tumour DNA (ctDNA) freely circulating into the bloodstream of cancer patients offers a unique opportunity to design new approaches for the non-invasive diagnosis and prognosis of a tumour. 

CTCs are tumour cells released into the blood from the primary tumour tissue. The detection of CTCs is challenging due to their low abundance in peripheral blood and intrinsic heterogeneity [109,110]. ctDNA is the small fraction of circulating cell-free DNA that is derived from tumour cells [111,112].

The identification of ctDNAs is today mostly performed using PCR-based methods including digital PCR [113,114,115] and next-generation sequencing platforms [116,117]. Such technologies are subject to limitations mainly associated with the PCR amplification. PCR is prone to sample contamination and tends to generate artefact fragments by recombination between homologous regions of DNA [118]. New approaches for the highly sensitive detection of ctDNAs are thus investigated to overcome limitations of currently available technologies. Biosensors offer attractive alternatives to presently available platforms, thanks to innovative possibilities for the sensitive, rapid and cheap detection of nucleic acid targets [12].

Plasmonic biosensors exploiting the peculiar properties of metal nanoparticles [20,119,120,121] have been used to design highly sensitive platforms for DNA detection [122,123], with specific applications using PNA probes to demonstrate the attomolar detection of point mutations in non-amplified human genomic DNA [124]. The coupling of plasmonic properties of AuNPs with PNA single-base mismatch recognition capacity obtained by conjugating PNA probes with AuNPs has been combined with anti-5-methylcytosine monoclonal antibody (mAb) capacity to detect methylated DNA for the simultaneous identification of both tumour-specific mutations of ctDNA and epigenetic modification (ctDNA methylation) within PIK3CA gene [40]. The assay uses AuNPs functionalized with 15-base long PNA probes with perfect matching for two hot spots in ctDNA (E542K and E545K). AuNPs functionalized with 5-methylcytosine monoclonal antibody (mAb) were used both to detect epigenetic modification in the ctDNA hybridized to the AuNPs–PNA system as well as to enhance the localised SPR shift measured after the adsorption of ctDNA on AuNPs–PNA. The mAb–AuNP enhancement allowed the detection of 50 fM solutions of ctDNA.

Pyrrolidinyl PNA is a conformationally rigid PNA derivative with a D-prolyl-2-aminocyclo-pentanecarboxylic acid (acpc) backbone [125]. acpcPNA exhibits a stronger directional preference for antiparallel binding and a higher affinity towards DNA over RNA than traditional PNA while keeping equal binding affinity and sequence selectivity. Human papillomavirus (HPV) type 16 DNA has been detected by combining anthraquinone (AQ)-labelled acpcPNA probes with the square-wave voltammetric biosensing [41]. acpcPNA–AQ probes were immobilized onto the chitosan layer of modified screen-printed carbon electrodes. The conformational change of the acpcPNA–AQ probe occurring upon the complementary DNA hybridization limited the electron transfer to the electrode surface capability of the AQ label, thus causing a decrease of the detected signal. A linear range in the 20 nM to 12 μM range was detected for the response of the assay with a LOD down to 4 nM. The test succeeded in identifying HPV type 16 DNA fragments in a PCR-amplified HPV infected cell line. When a similar detection scheme was applied by immobilising acpcPNA–AQ on graphene-polyaniline and using electrochemical impedance spectroscopy detection a linear range in the 10–200 nM range was obtained with LOD 2.3 nM [42]. In addition, in the latter case, the successful detection of PCR-amplified DNA from HPV type 16 positive SiHa cells was demonstrated.

The rare and complex nature of CTCs requires new tools to be developed for a smooth and detailed analysis of each cell [126]. In this context, the development of new platforms implementing the whole process from CTC capture to RNA or DNA detection is critical. The combination of microfluidics with voltammetric biosensing has been used to design a new platform integrating the capture of CTCs by antibody-modified magnetic nanoparticles, CTCs lyses and analysis of messenger RNA by voltammetric detection on nanostructured microelectrodes functionalized with PNA probes complementary to mRNA targets. The assay was successfully validated using samples collected directly from patient blood with a turn-around time of one hour useful to preserve properties of CTCs.

**Figure 2:**
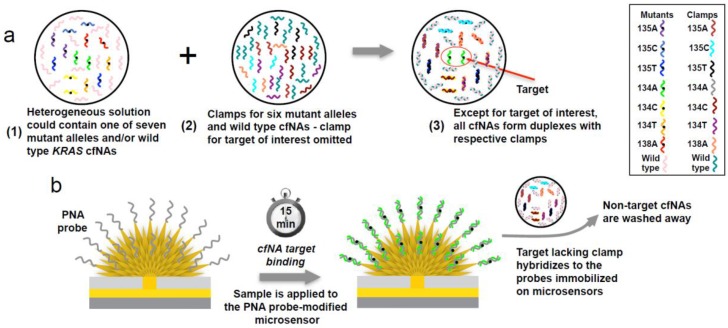
Schematic representation of the clamp assay used to detect KRAS mutations. (**a**) The sample (1) was mixed with PNA clamp sequences (2) to sequester the wild-type sequence and all of the mutated sequences different from the target KRAS sequence (3) (134A—green in the shown example). (**b**) The sample was then adsorbed onto the PNA probe-modified microelectrode and only the target KRAS sequence hybridized to the PNA probe. The other six mutants and wild-type nucleic acids were not able to bind and were washed away. Adapted with permission from Ref. [43].

A remarkable application of electrochemical biosensing to cancer diagnosis has been obtained by developing a voltammetric clamp assay for the screening of KRAS mutations in ctDNA from serum samples of cancer patients (Figure 2) [43]. A universal PNA probe complementary to the mutated KRAS gene target associated with lung, colorectal and ovarian cancers [127] has been immobilized on nanostructured gold microelectrodes. A mixture of PNA clamps was instead added to the human serum sample to hybridize sequences closely related to the target KRAS, thus favouring the interaction of the PNA probe with only the KRAS-mutated sequence. After KRAS target hybridization, the electrocatalytic reporter pair of [Ru(NH3)_6_]^3+^ and [Fe(CN)_6_]^3−^ was applied to read out the presence of target single-stranded ctDNA. More recently, an evolution of the assay has been proposed [44]. In this case, DNA clutch probes are used as ssDNA molecules to prevent the re-association of denatured ctDNA. The proposed electrochemical method can detect ctDNA within 30 min and displays excellent sensitivity and selectivity, being able to catch the target mutated allele at 1 fg μL^−1^ concentration in a background of wild-type alleles at concentration 100 pg μL^−1^. The detection of mutation in ctDNA obtained from the plasma of lung cancer and melanoma patients has been demonstrated using the above-described electrochemical biosensing approach.

The striking features of PNA help in better detection of SNPs linked to several types of cancer. In particular, recent applications of PNA biosensing have considered SNPs within KRAS or EGFR genes. Itogana et al. [128] recently demonstrated the rapid detection of KRAS mutations using Loop-Mediated Isothermal Amplification (LAMP) in combination with PNA clamp. LAMP amplification was performed in the presence of a PNA probe designed to clamp the KRAS gene wild-type sequence and LNA primers complementary to the mutated KRAS sequence. The LAMP amplification of wild-type KRAS DNA sequence was blocked by the PNA clamp, while the mutated KRAS was amplified within 50 min. 

PNA clamping is also useful to detect EGFR mutated gene in patients with non-small cell lung cancer [129]. In particular, PNA clamping combined with direct sequencing enables the detection of EGFR gene mutations in samples containing as few as 1% mutant alleles. 

## 3. Conclusions

The development of a sensitive, rapid, and robust bioanalytical platform for the detection of cancer-related DNA or RNA sequences is required to improve current possibilities for early cancer detection and patient follow-up. In this review, we summarised possibilities offered by PNA when used in combination with biosensing platforms for the sensitive discovery of nucleic acid biomarkers. Our purpose is to demonstrate how PNA probes used in biosensing can push down the selectivity and sensitivity of nucleic acid assays. The direct discrimination between closely related nucleic acid sequences can be achieved using PNA probes also in the presence of large non-target molecules, thus making available applications in cancer diagnostics.

In this review, we reported an overview of recent advances in the development of PNA-based biosensors with a particular emphasis on applications dealing with cancer diagnostics. The role of PNAs, when used in this specific domain, is discussed. Different established optical and electrochemical biosensors for the detection of clinically relevant DNAs and RNAs greatly benefit from PNA’s enhanced capability to detect sequences bringing point mutations. Electrochemical biosensors using PNA probes have been recently used to identify microRNA sequences. Results obtained propose such biosensors as promising platforms for the development of POC testing.

The combination of PNA probes and biosensors using nanostructured materials has been shown to improve the detection performances significantly. Most the advanced optical and electrochemical approaches here discussed take advantage of the neutral charge of PNA and exploit nanostructured materials to enhance the detected signal. Different biosensing platforms using PNA reaching fM sensitivity are already available and can identify both miRs as well as mutations in ctDNAs. The direct use of some of the described platforms on serum or plasma human sample has also been demonstrated.

Future perspectives in the field are linked to the ability to provide the final validation of some of the already described platforms in the clinical setting to demonstrate their performance under the most critical conditions.

## Figures and Tables

**Table 1:** Overview of PNA-based biosensors for the detection of RNA or DNA cancer biomarkers.

Target	Transduced Signal	LOD	Detection in Human Serum or Plasma	Reference
miR-21, miR-96 and miR-125b	Fluorescence	<1 pM	No	[31]
miR-21, miR-96 and miR-125b	Fluorescence	10 pM	No	[32]
miR-18a	Fluorescence	-	No	[33]
miR-21	Fluorescence	10 nM	No	[34]
miR-21	QCM	400 pM	Yes	[35]
miR-126, miR-182 and miR-152	Optical (Lateral flow test strip)	0.6 fM	No	[36]
miR-145	Electrochemical (Impedimetric and square-wave voltammetry)	0.37 fM	No	[37]
miR let-7a, let-7b, let-7c	Electrochemical (Impedimetric)	0.50 fM	No	[38]
miR let-7b, let-7c and miR 21	Electric (Graphene field-effect transistor)	<10 fM	Yes	[39]
E542K, E545K, methylation in PIK3CA gene	Optical (Localized surface plasmon resonance)	50 fM	Yes	[40]
HPV type 16 DNA, HPV types 18, 31 and 33	Electrochemical (Square-wave voltammetry)	4nM	No	[41]
DNA HPV type 16, type 18, type 31, and type 33	Electrochemical (Impedimetric)	2.3 nM	No	[42]
BRAF and KRAS DNA mutations	Electrochemical	1 fg μL^−1^	Yes	[43]
BRAF and KRAS DNA mutations	Electrochemical	1 fg μL^−1^	Yes	[44]
